# Supplementary material for: Endogenous opioid receptor system mediates costly altruism in the human brain
Source: Commun Biol. 2024 Oct 26;7:1401. doi: 10.1038/s42003-024-07084-7 (PMC11513155; doi:10.1038/s42003-024-07084-7)
Supplement: Supplementary file 1 — Supplementary information [file 42003_2024_7084_MOESM1_ESM.pdf]

Supplementary Materials

Stimuli

All videos played in the experiment (each video lasted 2s) were recorded in advance to span different shock levels (maximum to minimum: 6 to 2). All videos featured the same black background and black table. The participants could see the confederate's face and right hand, and the intensity of the electric shock could only be detected by changes in the facial expression, mainly by change of eye brows and mouth. The location of the hand was kept similar cross movies using a mark on the table that was covered by the hand, and hence invisible to the viewers of the movies. When shooting the videos, rather than giving a real electric shock to the hand, we poked the actor (author KS) on her thigh to induce her pain, as she reported this to be a more effective stimulus. Altogether, 17 of intensity 2, 12 of intensity 3, 9 of intensity 4, 7 of intensity 5 and 5 of intensity 6 were presented in the experiment randomly with fixed proportion in different intensity level. Pain levels were achieved by matching perceived pain levels to those used in Gallo et al. (2018) by author KI. Furthermore, there was significant positive relation between shock intensity in 1st video and donation indicating the stimuli worked well ($r = 0.672$, $p < 0.001$). For 1st video, the number of different shock intensity condition were similar, and reduced high shock intensity video in 2nd video caused by participants' donation, see **Supplementary Figure S1**.

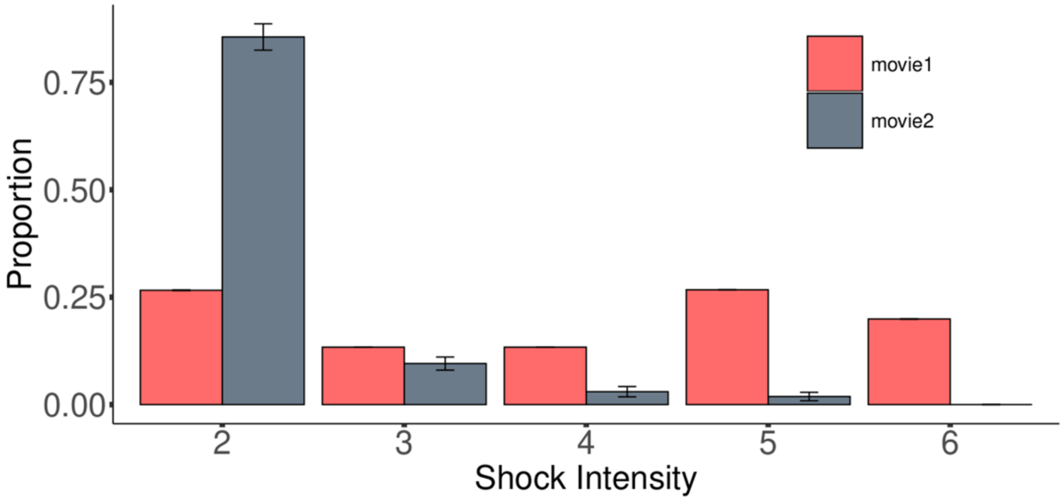

Figure S1. The proportion of shock intensity for 1st video and 2nd video. Error bar represents *SE*.

MOR-dependent responses to pain

Instead of using principal component analysis, we directly cumulate all the significate ROIs after corrected (**Fig. S2**).

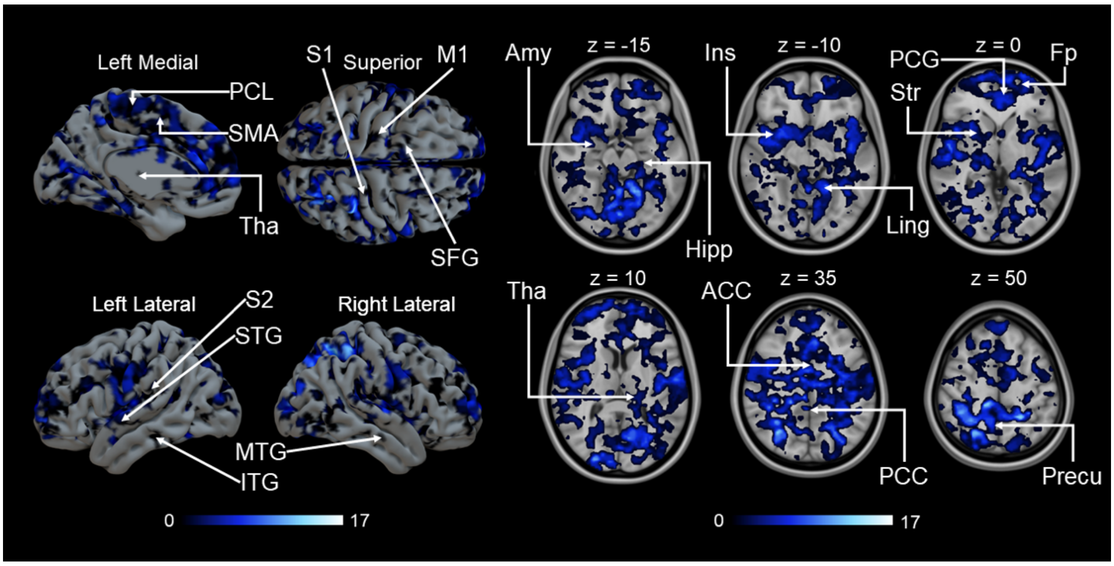

Figure S2. Cumulative maps showing the number of ROIs (out of 17) whose [^{11}C]carfentanil BP_{ND} was negatively correlated (p<0.05, FWE corrected at cluster level) with BOLD response to donation size. PCL = paracentral lobule, SMA = supplementary motor area, Tha = thalamus, S1 = primary somatosensory cortex, M1 = primary motor cortex, SFG = superior frontal gyrus, S2 = secondary somatosensory cortex, STG = superior temporal gyrus, MTG = middle temporal gyrus, ITG = inferior temporal gyrus, Amy = amygdala, Hipp = hippocampus, Ins = insula, Ling = lingual gyrus, PCG = paracingulate gyrus, Str = striatum, Fp = frontal pole, ACC = anterior cingulate cortex, PCC = posterior cingulate cortex, Precu = precuneous cortex.

Witnessing pain after costly helping

After donation, BOLD response to 2nd video was different from 1st video. Anterior insula, and angular gyrus were activated. The anterior insula in particular has been associated with vicarious pain activated in previous studies (**Fig. S3**).

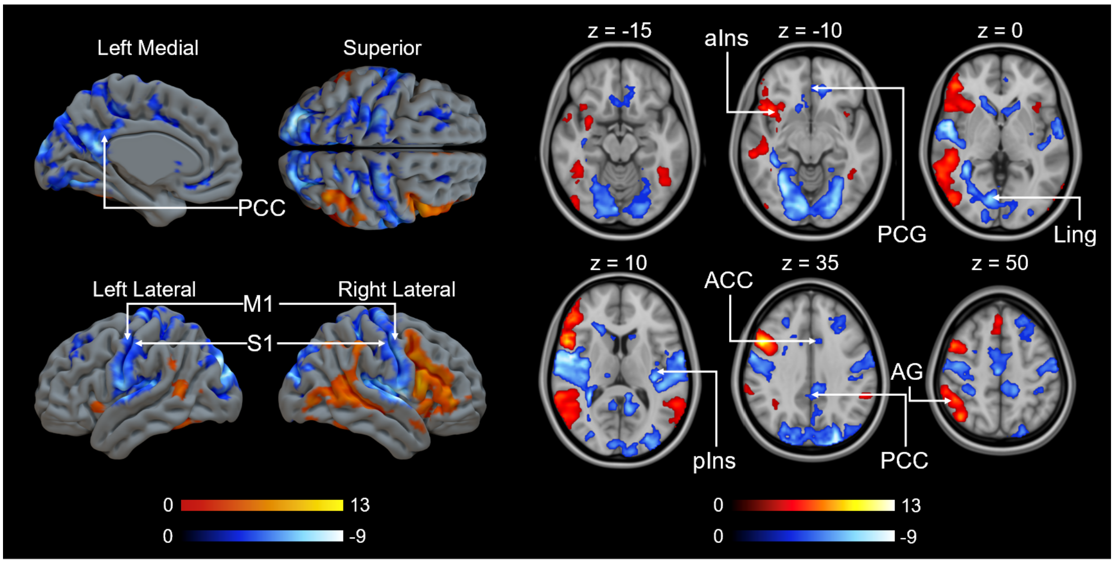

Figure S3. Main effect of pain perception in 2nd video. Colourbars indicate t statistic range. The data are thresholded at $p<0.001$, FWE corrected at the cluster level (Positive: $p_{unc}<0.001$, k=FWEc=110 voxels, $3.40<t<12.89$; Negative: $p_{unc}<0.001$, k=FWEc=121 voxels, $3.40<t<10.62$). PCC = posterior cingulate cortex, M1 = primary motor cortex, S1 = primary somatosensory cortex, aIns = anterior insula, PCG = paracingulate gyrus, Ling = lingual gyrus, pIns = posterior insula, ACC = anterior cingulate cortex, AG = angular gyrus.

Brain responses in donation phase

In donation phase, as shown in **figure S4**. Extensive brain areas were activated, including anterior insula, striatum, thalamus, anterior cingulate cortex, M1, S1.

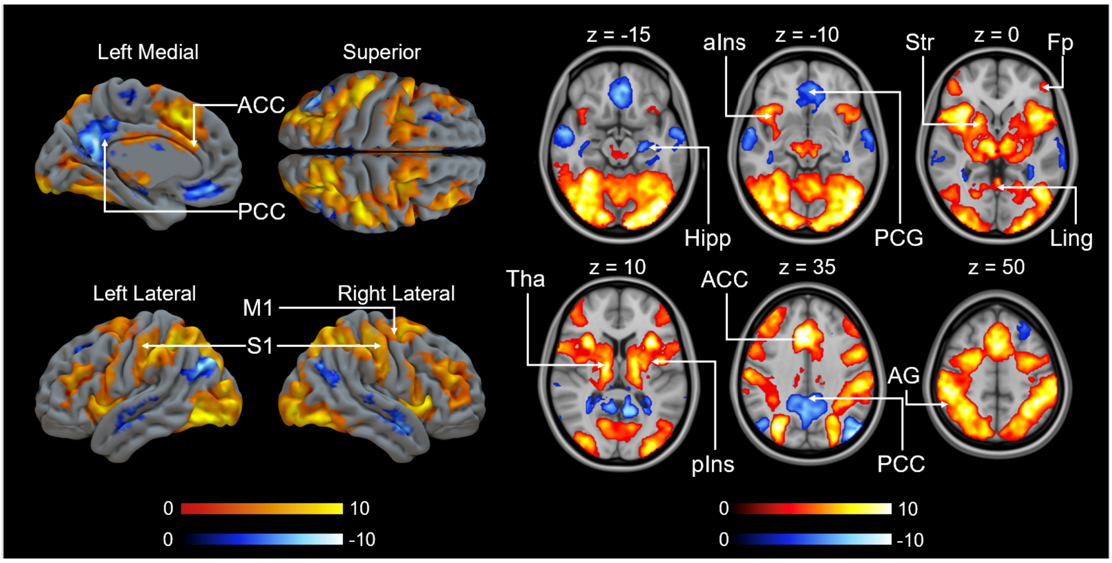

Figure S4. Main effect of brain activation to donation phase. Colourbars indicate t statistic range. The data are thresholded at $p<0.001$, FWE corrected at the cluster level (Positive: $p_{unc}<0.001$, k=FWEc=67990 voxels, $3.40<t<14.73$; Negative: $p_{unc}<0.001$, k=FWEc=221 voxels, $3.40<t<11.12$). ACC = anterior cingulate cortex, PCC = posterior

cingulate cortex, M1 = primary motor cortex, S1 = primary somatosensory cortex, Hipp = hippocampus, aIns = anterior insula, PCG = paracingulate gyrus, Str = striatum, Fp = frontal pole, Ling = lingual gyrus, Tha = thalamus, pIns = posterior, AG = angular gyrus.

Principal component analysis results

PCA result shows that the first three component can explain 90.1% of the variance, from the first component to the third component, they respectively explained 60.74%, 22.13%, and 7.27% of the variance. See the loadings of each ROIs below.

ROI	PC1	PC2	PC3
amy	0.317	0.632	-0.207
cau	0.149	0.121	-0.386
cer	0.231	0.254	-0.069
dacc	0.312	0.004	-0.024
inftemp	0.219	-0.010	0.152
ins	0.214	0.212	-0.091
midtemp	0.200	-0.042	0.091
nacc	0.547	0.040	0.607
ofc	0.248	-0.385	0.184
parsop	0.154	-0.218	-0.084
pcc	0.169	-0.224	-0.091
put	0.110	-0.248	-0.276
racc	0.230	-0.248	-0.178
supfront	0.120	-0.171	-0.210
suptemp	0.121	-0.188	-0.131
tempol	0.168	-0.197	-0.224
tha	0.239	-0.030	-0.355

Table S1. Loadings of the first three principal component on the ROIs. Amy = amygdala, cau = caudate, cer = cerebellum, dacc = dorsal anterior cingulate cortex, inftemp = inferior temporal gyrus, ins = insula, midtempo = middle temporal gyrus, nacc = nucleus accumbens, ofc = orbitofrontal cortex, parsop = pars opercularis, pcc = posterior cingulate cortex, put = putamen, racc = rostral anterior cingulate cortex, supfront = superior frontal gyrus, suptemp = superior temporal gyrus, tempol = temporal pole, tha = thalamus.

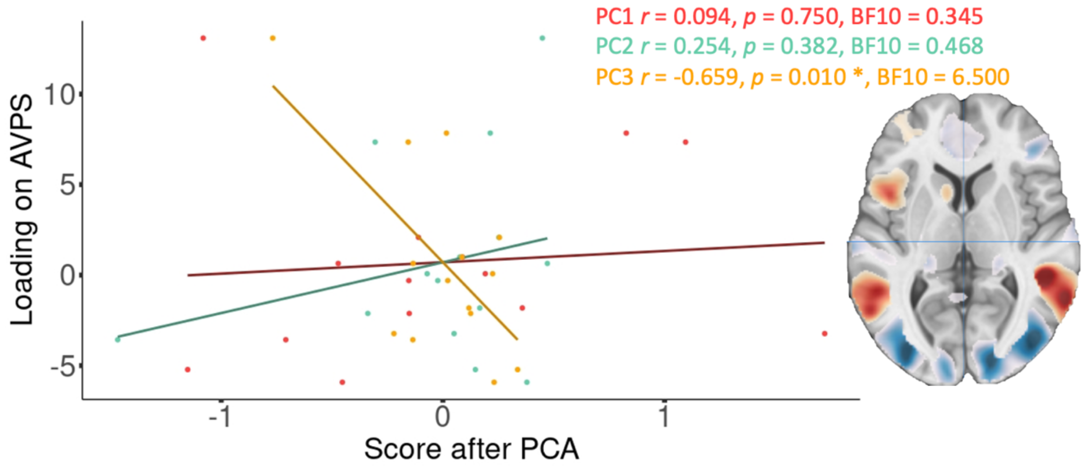

Figure S5. Correlation between the loading on AVPS of the parametric modulator donation size during 1st video and first three principal components. (Red=PC1, green=PC2, yellow=PC3). The sagittal slice on the right represents a thresholded version of the AVPS signature (https://identifiers.org/neurovault.collection:6332) that was dot-multiplied with each participants parametric modulator volume. Each dot represents the loading of one participant onto the AVPS.

Modulation effect of MOR availability to affective stress

To further explore whether MOR availability modulates affective stress when witnessing others in pain, we computed correlation between the contrast (1st video in the first session minus 1st video in the second session) and MOR availability and found positive correlation in caudate and ACC (Fig. S6). We also conducted separate analyses examining the relationship between MOR availability and BOLD response to the 1st video, utilizing donation size as a regressor. Interestingly, we observed similar results to those reported in the paper (Fig. 7) for the first session (Fig. S7). However, in the second session, no significant results were found. This once again suggests that MOR availability may play a role in reducing stress during the experiment.

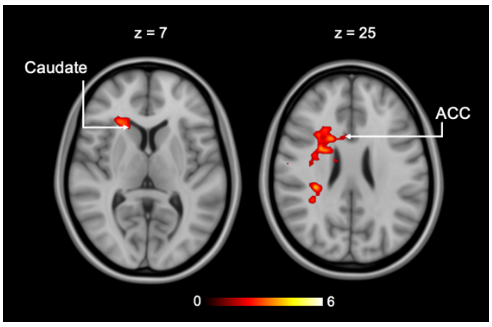

Figure S6. Positive correlation between the first component of MOR availability and the parametric modulator of BOLD response to donation size during the first session of 1st video subtracted from the second session. ACC = anterior cingulate cortex.

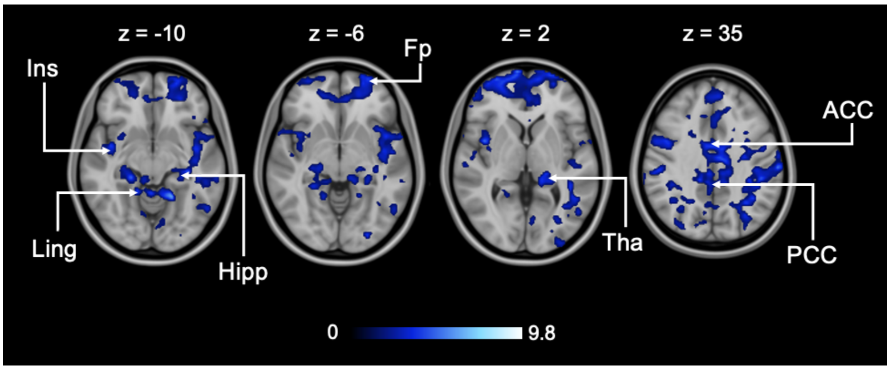

Figure S7. Negative correlation between the first component of MOR availability and the parametric modulator of BOLD response to donation size during 1st video in the first fMRI session. Ling = lingual gyrus, Hipp = hippocampus, ACC = anterior cingulate cortex, PCC = posterior cingulate cortex, Ins = insula, Fp = frontal pole, Tha = thalamus.

Witnessing pain effect from 1st video

We employed shock intensity as a regressor to predict brain activity of viewing 1st video (**Fig. S8**). The insula, thalamus, anterior cingulate cortex, and precuneus cortex—components of the empathy network—are more activated as shock intensity increases when observing others in distress. This finding suggests that the paradigm elicits emotional changes beyond video viewing.

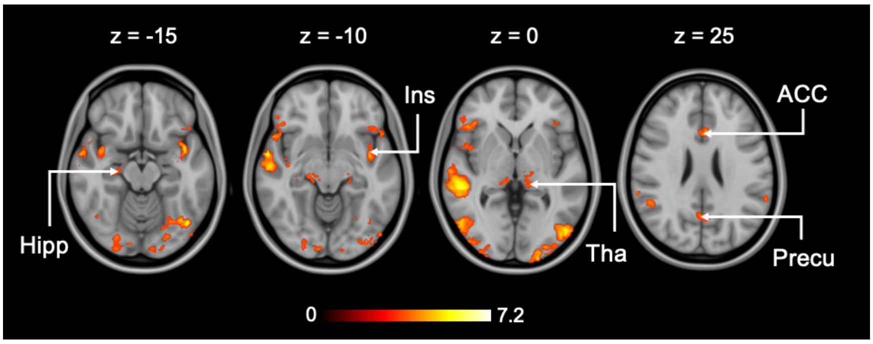

Figure S8. Brain regions with BOLD signals associated with shock intensity during the 1st video. Hipp = hippocampus, ACC = anterior cingulate cortex, Ins = insula, Tha = thalamus, Precu = precuneus cortex.

We used donation size as a regressor to explore the BOLD responses of seeing others in pain rather than shock intensity in the main text. Considering the inherent features of the paradigm, shock intensity and donation size are highly correlated, making it impractical to include both as regressors in the model. Shock intensity is predetermined before the experiment, while donation size reflects the participants' reactions and feelings. Therefore, we believe donation size is a better indicator of participants' feelings than shock intensity. As expected, the main results remain consistent between these two models.

Temporal influence on donation behavior

Upon incorporating time into our linear mixed model, we observed the following effects: The main effect of the 'trial' variable was found to be significant, β = -0.026, SE = 0.009, t = -2.920, p = 0.004). Additionally, we identified a significant interaction between 'trial' and shock intensity, β = 0.006, SE = 0.003, t = 2.095, p = 0.036). Furthermore, our analysis revealed that individuals tend to increase their donation behavior over time when watching videos of shock intensity 6 compared to shock intensity 2, β = 0.026, SE = 0.011, t = 2.305, p = 0.021) (see **Fig. S9**). It's worth noting that although there is a discernible change in subjects' donation behavior at shock intensity 6, this change is relatively small in practical terms and is unlikely to significantly impact the overall results.

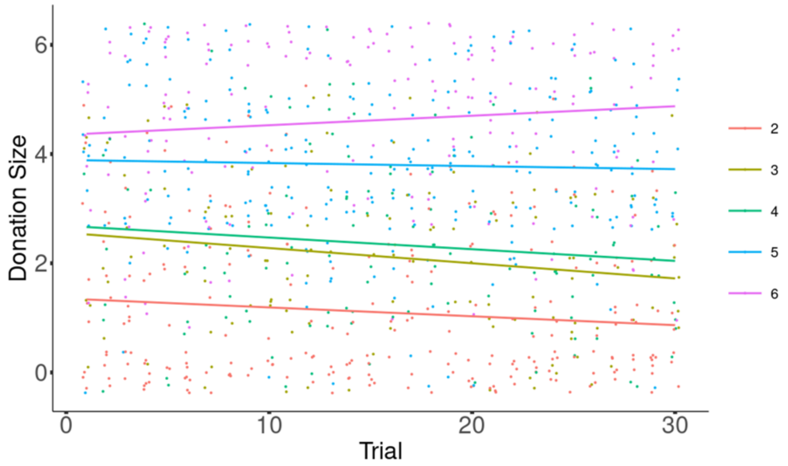

Figure S9. The plot illustrates how donation sizes change over time, with different colors representing varying shock intensities. Each line represents a regression line fitted to the data; each dot represents each trial.
