# Supplementary material for: Endogenous opioid receptor system mediates costly altruism in the human brain
Source: Commun Biol. 2024 Oct 26;7:1401. doi: 10.1038/s42003-024-07084-7 (PMC11513155; doi:10.1038/s42003-024-07084-7)
Supplement: Supplementary file 2 — Reporting summary [file 42003_2024_7084_MOESM2_ESM.pdf]

Corresponding author(s): COMMSBIO-24-0064B

Last updated by author(s): 2024.10.08

Reporting Summary

Nature Portfolio wishes to improve the reproducibility of the work that we publish. This form provides structure for consistency and transparency in reporting. For further information on Nature Portfolio policies, see our Editorial Policies and the Editorial Policy Checklist.

Statistics

For all statistical analyses, confirm that the following items are present in the figure legend, table legend, main text, or Methods section.

n/a	Confirmed
☐	☒ The exact sample size (*n*) for each experimental group/condition, given as a discrete number and unit of measurement
☐	☒ A statement on whether measurements were taken from distinct samples or whether the same sample was measured repeatedly
☐	☒ The statistical test(s) used AND whether they are one- or two-sided<br>*Only common tests should be described solely by name; describe more complex techniques in the Methods section.*
☐	☒ A description of all covariates tested
☐	☒ A description of any assumptions or corrections, such as tests of normality and adjustment for multiple comparisons
☐	☒ A full description of the statistical parameters including central tendency (e.g. means) or other basic estimates (e.g. regression coefficient) AND variation (e.g. standard deviation) or associated estimates of uncertainty (e.g. confidence intervals)
☐	☒ For null hypothesis testing, the test statistic (e.g. *F*, *t*, *r*) with confidence intervals, effect sizes, degrees of freedom and *P* value noted<br>*Give P values as exact values whenever suitable.*
☐	☒ For Bayesian analysis, information on the choice of priors and Markov chain Monte Carlo settings
☐	☒ For hierarchical and complex designs, identification of the appropriate level for tests and full reporting of outcomes
☐	☒ Estimates of effect sizes (e.g. Cohen's *d*, Pearson's *r*), indicating how they were calculated

Our web collection on statistics for biologists contains articles on many of the points above.

Software and code

Policy information about availability of computer code

Data collection	Data collected by self-wrote code with Presentation 23.0
Data analysis	Matlab 2021b, SPM12<br>R 4.1.3, Jasp

For manuscripts utilizing custom algorithms or software that are central to the research but not yet described in published literature, software must be made available to editors and reviewers. We strongly encourage code deposition in a community repository (e.g. GitHub). See the Nature Portfolio guidelines for submitting code & software for further information.

Data

Policy information about availability of data

All manuscripts must include a data availability statement. This statement should provide the following information, where applicable:

- Accession codes, unique identifiers, or web links for publicly available datasets
- A description of any restrictions on data availability
- For clinical datasets or third party data, please ensure that the statement adheres to our policy

Due to the hospital data confidentiality, the source data is not available. However, the group level results are accessible on NeuroVault (https://identifiers.org/neurovault.collection:14151)

Research involving human participants, their data, or biological material

Policy information about studies with human participants or human data. See also policy information about sex, gender (identity/presentation), and sexual orientation and race, ethnicity and racism.

Reporting on sex and gender	Sex was determined by self-report<br>Thirty healthy Finnish women (mean ± SD age: 24.7±5.65 years, range 19-42) with normal or corrected to normal vision were recruited in the study. To maximize statistical power of the study, only females were included because the MOR system shows a high level of sexual dimorphism (Kantonen, Karjalainen, Isoj, et al., 2020).
Reporting on race, ethnicity, or other socially relevant groupings	There is no race, ethnicity, or other socially relevant groupings information have been collected or used in this paper.
Population characteristics	See above
Recruitment	The subjects were recruited through university mailing lists and social media. Exclusion criteria included medications affecting the central nervous system, mood or anxiety disorders, psychotic disorders or neurological conditions, substance abuse and standard MRI and PET exclusion criteria.
Ethics oversight	The study was approved by the ethics board of the hospital district of Southwest Finland and conducted according to Good Clinical Practice and the Declaration of Helsinki (Approval number: 51/1801/2019).

Note that full information on the approval of the study protocol must also be provided in the manuscript.

Field-specific reporting

Please select the one below that is the best fit for your research. If you are not sure, read the appropriate sections before making your selection.

☒ Life sciences ☐ Behavioural & social sciences ☐ Ecological, evolutionary & environmental sciences

For a reference copy of the document with all sections, see nature.com/documents/nr-reporting-summary-flat.pdf

Life sciences study design

All studies must disclose on these points even when the disclosure is negative.

Sample size	Based on previous PET-fMRI studies (e.g. Nummermaa et al. 2018) we expected effect size of .8 giving us a statistical power of .88 with an N of 14 (calculated with the 'pwr' package in R)<br>Nummenmaa, L., Saanijoki, T., Tuominen, L. et al. μ-opioid receptor system mediates reward processing in humans. Nat Commun 9, 1500 (2018). https://doi.org/10.1038/s41467-018-03848-y
Data exclusions	No data was excluded.
Replication	Piloting, robust statistical methods, adequate sample size and consistent results with previous studies.
Randomization	The were no experimental groups and thus no need for randomization.
Blinding	The were no experimental groups and thus no need for blinding.

Reporting for specific materials, systems and methods

We require information from authors about some types of materials, experimental systems and methods used in many studies. Here, indicate whether each material, system or method listed is relevant to your study. If you are not sure if a list item applies to your research, read the appropriate section before selecting a response.

Materials & experimental systems

n/a	Involved in the study
☒	☐ Antibodies
☒	☐ Eukaryotic cell lines
☒	☐ Palaeontology and archaeology
☒	☐ Animals and other organisms
☒	☐ Clinical data
☒	☐ Dual use research of concern
☒	☐ Plants

Methods

n/a	Involved in the study
☒	☐ ChIP-seq
☒	☐ Flow cytometry
☐	☒ MRI-based neuroimaging

Plants

Seed stocks

Report on the source of all seed stocks or other plant material used. If applicable, state the seed stock centre and catalogue number. If plant specimens were collected from the field, describe the collection location, date and sampling procedures.

Novel plant genotypes

Describe the methods by which all novel plant genotypes were produced. This includes those generated by transgenic approaches, gene editing, chemical/radiation-based mutagenesis and hybridization. For transgenic lines, describe the transformation method, the number of independent lines analyzed and the generation upon which experiments were performed. For gene-edited lines, describe the editor used, the endogenous sequence targeted for editing, the targeting guide RNA sequence (if applicable) and how the editor was applied.

Authentication

Describe any authentication procedures for each seed stock used or novel genotype generated. Describe any experiments used to assess the effect of a mutation and, where applicable, how potential secondary effects (e.g. second site T-DNA insertions, mosiacism, off-target gene editing) were examined.

Magnetic resonance imaging

Experimental design

Design type

Block design

Design specifications

There were two imaging runs with 15 trials in each for each participant. In each run, there is only one block. Each trial is between 13.5s and 35s second. There is 8-13s between each trial.

Behavioral performance measures

We collected how much participants donated. Linear mixed model was used to establish participants' performance.

Acquisition

Imaging type(s)

Structural and functional images

Field strength

3

Sequence & imaging parameters

echo-planner imaging (EPI) sequence (45 slices; slice thickness = 3 mm; TR = 2600 ms; TE = 30 ms; flip angle =75°; FOV = 24 mm; voxel size = 3*3*3 mm3. Axial (transverse) orientation.

Area of acquisition

Whole brain

Diffusion MRI ☐ Used ☒ Not used

Preprocessing

Preprocessing software

fMRIPprep 1.3.0.post2

Normalization

spatial normalization to the ICBM 152 Nonlinear Asymmetrical template version 2009c using nonlinear registration with antsRegistration (ANTs 2.2.0)

Normalization template

ICBM 152 Nonlinear Asymmetrical template version 2009c

Noise and artifact removal

automatic removal of motion artifacts using ICA-AROMA.

Volume censoring

We did not perform volume cencoring

Statistical modeling & inference

Model type and settings

fMRI data were analyzed in SPM12 (Wellcome Trust Center for Imaging, London, UK, https://www.fil.ion.ucl.ac.uk/spm/) (N = 30). To investigate regions activated by i) seeing pain in the 1st video, ii) donating, and iii) seeing pain in the 2nd video, first-

level general linear models (GLM) were estimated by modeling the 1st video, donation phase, and the 2nd video by using boxcar regressors convolved with HRF in the design matrix. Donation size (trial-wise donations for each subject) was entered as parametric modulator for the 1st video. Subject-wise contrast images were then generated for main effects of 1st video, donation phase, 2nd video. Additionally, a subtraction contrast was computed for 1st vs. 2nd video. The contrast images were then subjected to second-level (random effects) analysis. Results are shown after FWE correction for cluster-size, by initially thresholding statistical maps at punc<0.001, identifying the FWEc minimum cluster-size value for FWE correction at the cluster-size level, and then thresholding the statistical maps again at punc<0.001 and k=FWEc-1.
Three approaches were taken to characterize the interactions between MOR availability and BOLD responses in pain perception and costly altruism (N = 14). In the first two approaches, a principal component analysis (PCA) was used to reduce the dimensionality in the BPND values across our ROIs. This was done because regional MOR availability has high autocorrelation 68, thus PCA would increase the power of our analyses by reducing the multiple comparison correction that would otherwise reduce power. We found the first 3 PCs to explain >90% of the variance, with 61%, 22% and 7% of variance explained, respectively. To identify voxels with BOLD responses that depend on individual differences in the overall MOR signal, we used the first PC to predict the voxel-wise BOLD responses to the 1st video with donation size as a parametric modulator and the donation phase, separately. Specifically, we used the same parametric model as the fMRI analysis in the first-level model, and input the first PC in the second-level model for 1st video and donation, in separate models. Second, to explore the relationship between individual MOR differences and responses in the vicarious pain observation network, we used the affective vicarious pain signature (AVPS) to dot-multiply the 1st level beta maps to 1st video with donation size as a parametric regressor for each participant 69, thereby reducing each parameter estimate volume to a scalar value, and computed the correlation between the resulting value and the first 3 MOR PCs. Finally, to replicate previous studies on the links between MOR availability and haemodynamic responses to vicarious pain and arousal 48,67, the voxel-wise BOLD responses to donation size in 1st video were predicted with ROI-wise [11C]carfentanil binding potentials using whole-brain linear regression analysis with a statistical threshold set at $p < 0.05$, FWE-corrected at cluster-level. We then computed a cumulative map of the binarized MOR × BOLD beta maps to highlight regions whose BOLD responses were most consistently dependent on regional MOR availability.

Effect(s) tested

Main effect of brain response to the first video. Parametric model effect of donation size. Contrast effect between first and second video. Effect of regressor of MOR availability

Specify type of analysis: ☒ Whole brain ☐ ROI-based ☐ Both

Statistic type for inference
(See Eklund et al. 2016)

voxel-wise in fMRI analysis, ROI-wise in fMRI-PET analysis

Correction

fMRI analysis: FWE correction for cluster-size, by initially thresholding statistical maps at punc<0.001.fMRI-PET analysis: statistical threshold set at $p < 0.05$, FWE-corrected at cluster-level.

Models & analysis

n/a	Involved in the study
☒	☐ Functional and/or effective connectivity
☒	☐ Graph analysis
☒	☐ Multivariate modeling or predictive analysis
